# Comparative transcriptome analysis of *Eimeria maxima* (Apicomplexa: Eimeriidae) suggests DNA replication activities correlating with its fecundity

**DOI:** 10.1186/s12864-018-5090-2

**Published:** 2018-09-24

**Authors:** Dandan Hu, Chaoyue Wang, Si Wang, Xinming Tang, Chunhui Duan, Sixin Zhang, Jinxia Suo, Miner Deng, Yanli Lv, Xun Suo, Xianyong Liu

**Affiliations:** 1 State Key Laboratory for Agrobiotechnology, China Agricultural University, Beijing, China; 2 Key Laboratory of Animal Epidemiology and Zoonosis of Ministry of Agriculture, College of Veterinary Medicine, China Agricultural University, Beijing, China; 3 National Animal Protozoa Laboratory, College of Veterinary Medicine, China Agricultural University, Beijing, China

**Keywords:** *Eimeria maxima*, Precocious line, Transcriptome, Fecundity

## Abstract

**Background:**

Chicken coccidiosis, caused by the infection of *Eimeria* species, leads to important economic losses to the poultry industry. Vaccination with attenuated live parasites seems to be the best way to control this disease. Attenuated eimerian parasites with shortened prepatent times show great changes in intracellular development compared to their parent strains but the mechanisms involved in these biological differences are still unclear.

**Results:**

In this study, we obtained a precocious line of *E. maxima* by sequential selection of 22 generations of early shed oocysts in chickens and performed a comparative transcriptome analysis of three different developmental stages of the precocious line and its parent strain using Illumina high-throughput sequencing. Our *E. maxima* precocious line showed decreased pathogenicity, reduced fecundity and a greatly shorted prepatent time of only 98 h. We found that typical gene changes in the stage development from unsporulated to sporulated oocyst and from sporulated oocyst to merozoite were marked by upregulated organelle genes and protein translation related genes, respectively. Additionally, major differences between the precocious line and its parent strain were detected in the merozoite stage, characterized by downregulated genes involved in protein cleavage and DNA replication activities.

**Conclusions:**

Our study generated and characterized an *E. maxima* precocious line, illustrating gene expression landscapes during parasite development by transcriptome analysis. We also show that the suppressed DNA replication progress in the merozoite stage in the precocious line may result in its reduced fecundity. These results provide the basis for a better understanding of the mechanism of precocity in *Eimeria* species, which can be useful in studies in early gametocytogenesis in apicomplexan parasites.

**Electronic supplementary material:**

The online version of this article (10.1186/s12864-018-5090-2) contains supplementary material, which is available to authorized users.

## Background

*Eimeria* species, belonging to Apicomplexan parasites as *Toxoplasma* and *Plasmodium*, causes chicken coccidiosis in almost all poultry farms worldwide [1]. Coccidiosis leads to hematochezia (even death) and lower feed conversion rate in chicken, resulting in more than $3 billion USD annual economic losses for the poultry industry [1–4]. In the past decades, anticoccidial drugs have been predominantly used in the control of chicken coccidiosis [5–7]. However, due to the resistance to anticoccidial drugs in *Eimeria* field strains, and the legislative and consumer pressure on reducing drug use throughout the food chain, the discovery and use of alternative methods for coccidiosis control has become of greater importance [5, 7].

Attenuated live vaccines, composed of different precocious *Eimeria* species and strains are simple to apply, cost effective and a safe method against coccidiosis [8, 9]. To generate attenuated *Eimeria* strains, chickens are orally infected with sporulated oocysts. Released sporozoites invade intestinal epithelial cells (IECs) and undergo a serial asexual amplification (schizogony), generating large number of merozoites. The last generation merozoites then develop into sexual microgametes or macrogametes, which fuse to form zygotes, which then mature as unsporulated oocysts and are released in the droppings of the chickens to sporulate in the external environment. By successive selection of early shed oocysts, precocious lines can be established. These *Eimeria* precocious lines are characterized by greatly reduced pathogenicity and prepatent time, and have faster asexual stages or deletion of some schizonts generations, and thus generate fewer merozoites and oocysts [10–16]. In the case of *E. maxima*, the Weybridge strain was attenuated by selection for early maturation of oocysts during a 13-serial passage [15]. The precocious line reduced prepatent times from ~ 120 h to less than 107 h, showing less reproduction and pathogenicity with equivalent immunogenicity.

To understand the molecular basis behind this special phenotype in *Eimeria* species, Shirley and Harvey created a genetic linkage map for *E. tenella* by crossbreeding a precocious strain with an arprinocid-resistant strain, and mapped the trait of precocious development to chromosome 2, but the sequences and genes in this region remained unknown [17]. Recently, a transcriptional comparison between the virulent and precocious strains of an *E. tenella* Nippon strain was carried out [18]; however, the genetic mechanism of “precocity” in *Eimeria* species remains unknown.

In this study, we first generated and characterized a precocious line of *E. maxima* Beijing strain with a prepatent time of only 98 h. We then performed a comparative transcriptional analysis between different developmental stages of the parent strain and the precocious line to show the mRNA landscape of parasite development and precocity. Our work provides a general knowledge for researchers in studying “precocity” in *Eimeria* species, and fundamental data for studies in other eimerian parasites.

## Methods

### Animals and parasites

One- to six-week-old AA broilers, used for passages and precocious line selections, were purchased from Beijing Arbor Acres Poultry Breeding (Beijing, China). Two-week-old SPF chickens, used in immunological studies, were purchased from Merial Animal Health Co., Ltd. (Beijing, China). All birds were fed with a coccidia-free diet and water ad libitum. The wild type *E. maxima* Beijing strain (BJ-WT-130) has been maintained in our laboratory [19]. The *E. maxima* Shandong and Hebei strains were isolated by infection with a single oocyst derived from poultry farms in Shandong Province and Hebei Province in China, respectively. The procedures for collection, purification and sporulation of the parasite were carried out as previously described [20]. Cervical dislocation was used for chickens necessary for sacrifice, which resulting in rapid and painless loss of consciousness.

### Precocious line selection and characterization

The *E. maxima* precocious line Beijing strain (BJ-PL-98) was selected as previously described [15]. Briefly, AA broilers were inoculated with oocysts produced in the first few hours of a patent infection. Details for the successive selection are listed in Additional file 1.

Comparative studies of endogenous development, reproductivity and immunogenicity between BJ-PL-98 and BJ-WT-130 were performed with modifications to previously described methods [15, 21, 22]. Sections of small intestine of chickens after 32, 64, 80, 88, 96, 104, 112, 120, 128, 140 and 150 h post-inoculation were checked after hematoxylin and eosin staining (H&E). The reproductivity of BJ-PL-98 and BJ-WT-130 was estimated by oocyst output counting (mean of three independent counts) of two groups of chickens infected with 5000 oocysts/bird for each strain or line. To compare the pathogenicity of the parent strain and the precocious line, six groups of SPF chickens (three per group) were dosed with 10^4^, 1 × 10^5^ and 5 × 10^5^ oocysts of BJ-PL-98 or BJ-WT-130, respectively. An uninfected control group was also set. Body weight gain of each bird was monitored at 0, 7 and 14 days post inoculation (dpi).

For immunogenicity studies, SPF chickens were immunized with either 200 or 500 BJ-PL-98 or BJ-WT-130 oocysts, and then challenged with BJ-WT-130 (1 × 10^5^ oocysts/bird) two weeks later. The *E. maxima* Shandong and Hebei strains were used in cross-immune protection tests, in which chickens were immunized with 200 BJ-PL-98 oocysts and separately challenged with 1 × 10^5^ oocysts from the Shandong or Hebei strains. Unimmunized & unchallenged (UUC) and unimmunized & challenged (UC) groups were set as controls. Total oocyst output was measured at 14 and 28 days post first inoculation.

### Preparation of samples for RNA-Seq

For RNA sequencing, unsporulated oocysts (two biological replicates), sporulated oocysts (two biological replicates) and merozoites (two/three biological replicates) of BJ-PL-98 and BJ-WT-130 were isolated as described previously [23]. Briefly, birds were sacrificed at six and seven days post inoculation of BJ-PL-98 and BJ-WT-130, respectively. Afterwards, minced small intestine was digested with 2.5% trypsin (Sigma, MO, USA), and filtered to separate unsporulated oocysts. Sporulated oocysts were collected after an additional 72 h of sporulation at 28 °C. According to our endogenous study, the merozoites of fourth generation schizonts of BJ-PL-98 and BJ-WT-130 were collected at 88 and 120 h after inoculation, respectively. Briefly, the intestines were cut into small pieces and incubated at 42 °C for 20–30 min in digestion buffer (Hanks BS + 10 mM MgCL_2_ + 0.25% trypsin + 1% taurocholic acid), and then merozoites were washed, filtered and finally purified with a DE-52 column. All samples were stored in liquid nitrogen immediately after collection.

### RNA extraction, libraries preparation and RNA-Seq

Total RNAs were isolated using Trizol® (Life Technologies, MD, USA), and genomic DNA was digested with DNase I (Qiagen, Hilden, Germany). Purity, concentration, and integrity of the RNAs were tested using NanoPhotometer® (IMPLEN, CA, USA), Qubit® RNA Assay Kit in Qubit® 2.0 Fluorometer (Life Technologies, CA, USA) and RNA Nano 6000 Assay Kit of the Bioanalyzer 2100 system (Agilent Technologies, CA, USA), respectively. Only qualified samples were used for libraries preparation. Sequencing libraries were generated using NEBNext® Ultra™ RNA Library Prep Kit for Illumina® (NEB, USA) following manufacturer’s recommendations. Illumina Hiseq X Ten platform was employed for sequencing, with 150 bp paired-end reads generated.

Genome and gene-models of *E. maxima* in ToxoDB release-35 [24] were downloaded for analysis. Index of the reference genome was built using Bowtie v2.2.3 and paired-end clean reads were aligned to the reference genome using TopHat v2.0.12. [25]. HTSeq v0.6.1 [26] was used to count the reads numbers mapped to each gene. Differential expression analysis of two conditions was performed using the DEGSeq2 R package [27]. *P*-values were adjusted using the Benjamini & Hochberg method. Corrected *p*-values < 0.05 and log_2_ (fold change) > 1 were regarded as significantly different. The FPKM value of each gene was calculated as the formula described before (“FPKM = 10^6^ × C/ (N × L)” where C is the number of paired-reads mapped to the exon model of a gene, N is the total number of mapped reads in each library and L is the length of the gene in kilobases) [28]. The FPKM values of selected genes were used for drawing of cluster with the R package pheatmap based on Euclidean distance and complete clustering [29]. Gene Ontology (GO) enrichment analysis and Kyoto Encyclopedia of Genes and Genomes (KEGG) enrichment analysis of differentially expressed genes was implemented by the GOseq R package [30] and KOBAS [31] software using default parameters.

### qPCR

To validate the RNA-Seq data, we selected six differently expressed genes for qPCR experiments. The cDNA samples were synthesized from DNase-treated RNAs employed in the RNA-Seq using TransScript One-Step gDNA Removal and cDNA Synthesis SuperMix (Transgen Biotech, Beijing). PCR reactions were performed on a 7500 Real-Time PCR System (Applied Biosystems) using SYBR Premix Ex Taq Tli RNaseH Plus (ROX) (Takara, Beijing). For each sample, reactions were performed in three replicates. The primers are listed in the Additional file 2. The expression of each gene was normalized to the reference gene glyceraldehyde 3-phosphate dehydrogenase (GAPDH) as reported previously [28].

### Statistical analysis

Unpaired two-tailed Student’s t-tests were used in oocyst output, average body weight gain and qPCR calculations using GraphPad Prism® Version 6.03 (GraphPad Software Inc., USA).

## Results

### Selection and endogenous development observation of the precocious line BJ-PL-98

For the selection of the precocious line, we performed serial passages of *E. maxima* in chicken. After 20 generations, the prepatent time decreased from 130 to 108 h. After 2 additional selections the precocious line oocysts could be detected in the droppings 98 h post-infection (hpi) (Additional file 1). This prepatent time was found to be stable as verified in the following three (or more) generations (Additional file 1). This resulted in an *E. maxima* precocious line (BJ-PL-98) with a prepatent time 32 h shorter than the parent strain.

**Fig. 1:**
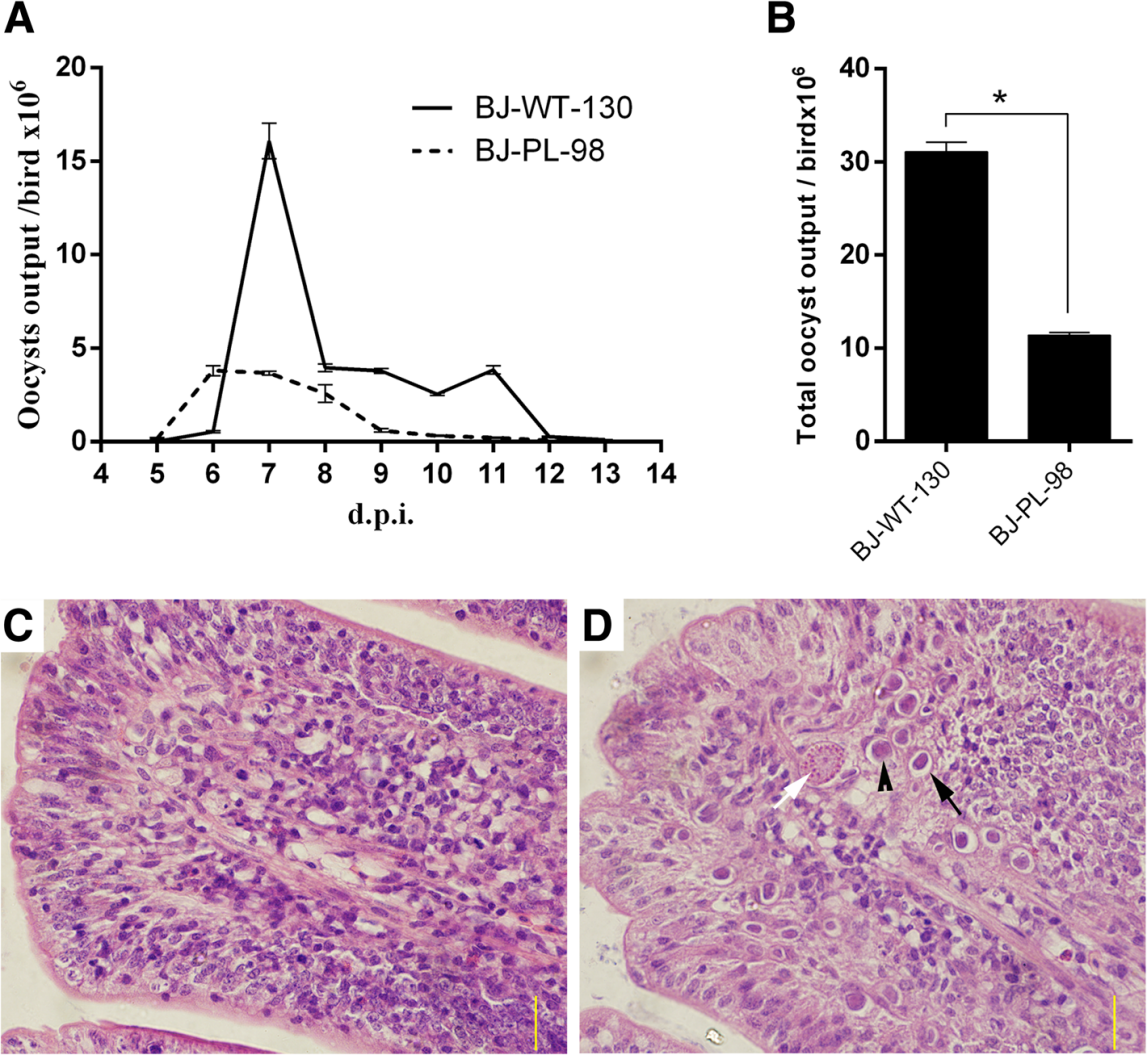
Oocyst output and endogenous development of *E. maxima* precocious line BJ-PL-98. **a**: oocyst output curves for the *E. maxima* precocious line (BJ-PL-98) and its parent strain (BJ-WT-130). Chickens were inoculated with 5000 oocysts/bird of either BJ-PL-98 or BJ-WT-130. Oocysts yields from days four to thirteen post-inoculation were measured every single day. (**b**) Total oocyst output of BJ-PL-98 and BJ-WT-130 were calculated by sampling and counting in three measurements. Endogenous development of BJ-WT-130 (**c**) and BJ-PL-98 (**d**) 96 hpi were observed by H&E straining of intestine sections. The black arrow shows the schizont, the white arrow shows the mature gametocyte and the black arrow head shows the microgametocyte. Asterisk indicates *p* < 0.001. Bar = 50 μm

The oocyst output peak of BJ-PL-98 occurred approximately six dpi, clearly different from that from its parent strain BJ-WT-130 (peak at seven dpi) (Fig. 1a). Total oocyst output of BJ-PL-98 was reduced 63.3% compared to BJ-WT-130, indicating weaker reproductivity (Fig. 1b). The first generation schizogony of BJ-PL-98 and BJ-WT-130 could be detected in the glandular epithelial cells in both the duodenum and the jejunum 32 hpi, but the biggest number of schizonts emerged in villus cells in the jejunum 88 hpi and 120 hpi, respectively (Additional file 3). For BJ-PL-98, mature gametocytes were first found 96 hpi (Fig. 1c, d), and reached a peak 120 hpi, while unsporulated oocysts were found 104 hpi (Additional file 3). For the BJ-WT-130, mature gametocytes were first detected 128 hpi, reaching its highest number 150 hpi. A small number of unsporulated oocysts could be detected 140 hpi (Additional file 3).

### Pathogenicity and immunogenicity of BJ-PL-98

**Fig. 2:**
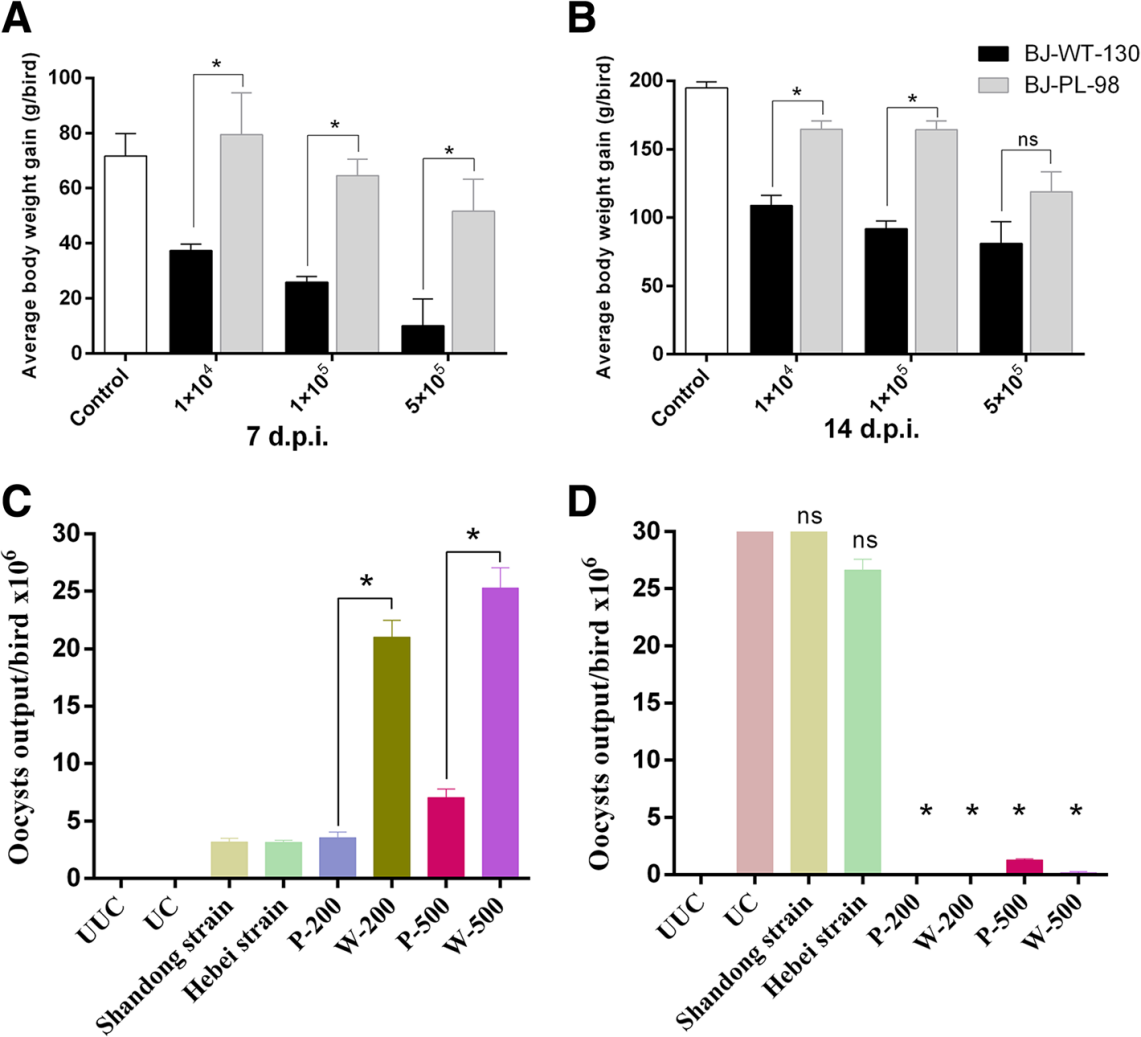
Pathogenicity and immunogenicity of the *E. maxima* precocious line BJ-PL-98. Average body weight gains of birds (*n* = 3) were measured 7 (**a**) and 14 (**b**) days after inoculation with three different doses of BJ-PL-98 and BJ-WT-130. The total oocyst output of birds was measured after immunization (**c**) and challenge (**d**) with different doses and strains of *E. maxima*. UUC: unimmunized and unchallenged; UC: unimmunized but challenged with BJ-WT-130 (1 × 10^5^ oocysts/bird); Shandong strain group, Hebei strain group and P-200 group: immunized with BJ-PL-98 (200 oocysts/bird), and challenged with 1 × 10^5^ oocysts/bird using Shandong strain, Hebei strain and BJ-WT-130, respectively. P-500 group: immunized with BJ-PL-98 (500 oocysts/bird) and challenged with BJ-WT-130 (1 × 10^5^ oocysts/bird). W-200 and W-500 groups: birds were immunized with 200 and 500 BJ-WT-130 oocysts each, respectively, and challenged with BJ-WT-130 (1 × 10^5^ oocysts/bird). Asterisks represent *p* < 0.05, ns means not statistically significant (*p* > 0.05)

To compare the pathogenicity of the BJ-PL-98 and BJ-WT-130, we measured the average body weight gain of birds after inoculation with varied doses of BJ-PL-98 and BJ-WT-130. Average body weight gains showed a gradual decrease as the inoculation dose increased (Fig. 2a, b). When given equal doses, average body weight gain of birds inoculated with BJ-PL-98 was significantly higher than with BJ-WT-130 (except in the 5 × 10^5^ group 14 dpi) (Fig. 2a, b), which reflected the fact that BJ-PL-98 has lower pathogenicity.

For immunogenicity comparison tests, birds were immunized with BJ-PL-98 or BJ-WT-130 and then challenged with BJ-WT-130. The total oocyst output of each immunized group showed very significant decreases compared to the unimmunized group (Fig. 2c, d). This result suggests that 200 BJ-PL-98 oocysts could provide strong protection against the wild strain since the immunogenicity of BJ-PL-98 did not change after sequential selection with more than 25 generations. On the other hand, oocyst outputs of field strains could not be significantly reduced by immunization with BJ-PL-98, which indicated that BJ-PL-98 could not provide cross-protection to different field strains (Fig. 2d).

### Gene expression signature during parasite developmental stage transition

To get a complete view of gene expression landscapes during *E. maxima* development, we performed RNA-Seq analysis of three developmental stages of *E. maxima*. Sequencing data were filtered for host contamination and mapped to *E. maxima* genome (Additional file 4), and different expressed genes were analyzed (Additional file 5). Expression of six genes in each sample was validated by qPCR (see Additional file 6)*.*

In the comparison between unsporulated oocysts and sporulated oocysts, we found 2387 genes upregulated and 2819 genes downregulated in BJ-WT-130, and 3163 genes upregulated and 3407 genes downregulated in BJ-PL-98 (Additional file 7). For both BJ-WT-130 and BJ-PL-98, we found 30 more highly transcribed organelle genes in sporulated oocysts than in the unsporulated oocysts (Additional file 8), including microneme (MICs), rhoptry (ROPs) and inner membrane complex (IMCs). Because the dense granule (GRA) genes of *Eimeria* species were not well annotated, we only found one upregulated GRA (EMWEY_00005390, annotated as GRA10). These results were consistent with the biological process during oocyst sporulating, when one diploid unsporulated oocyst divided into eight haploid sporozoites, and subcellular organelles emerged. Additionally, we found that 21 surface antigen genes (SAGs) were also strongly upregulated (Additional file 8).

**Fig. 3:**
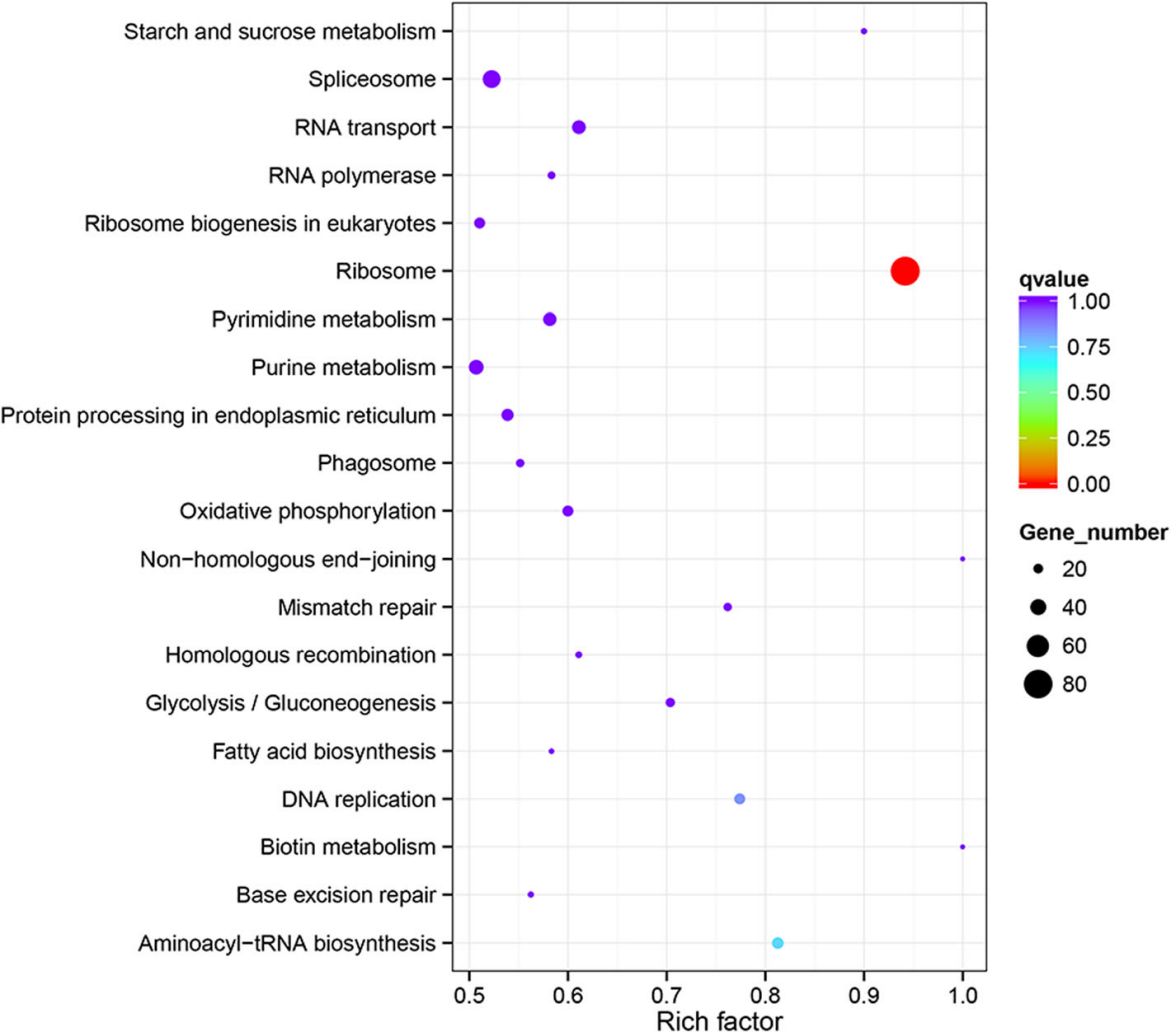
KEGG pathway enrichment for DEGs between sporulated oocysts and merozoites. The DEGs between sporulated oocysts and merozoites of BJ-WT-130 were analyzed for KEGG pathway enrichment using hypergeometric tests / Fisher’s exact tests. Benjamini and Hochberg tests were used for false discovery rate (FDR) correction

When comparing sporulated oocysts with merozoites, we found 2918 genes upregulated and 2960 genes downregulated in BJ-WT-130, and 2156 genes upregulated and 2582 genes downregulated in BJ-PL-98. These differentially expressed genes (DEGs) were significantly enriched (corrected *p* value < 0.05) in protein translation related gene ontology (GO) terms, including translation, peptide biosynthetic process, amide biosynthetic process, peptide metabolic process and cellular amide metabolic process. KEGG enrichment analysis indicated that the exact pathway involved in these GO terms was “Ribosome” (corrected p value < 0.05) (Fig. 3), which suggested a more active protein expression in the merozoite stage.

### Transcriptional basis for precocity in *E. maxima*

To explore the potential mechanism involved in the unique “precocity” phenomenon in *Eimeria* species, we performed a stage-by-stage comparison between the parent strain and the precocious line of *E. maxima*. In the unsporulated oocyst stage, only 860 DEGs were detected (551 upregulated in the precocious line, Additional file 8), and since these genes were poorly annotated (63% of them were annotated as hypothetical protein), this limited us in mining further information.

**Fig. 4:**
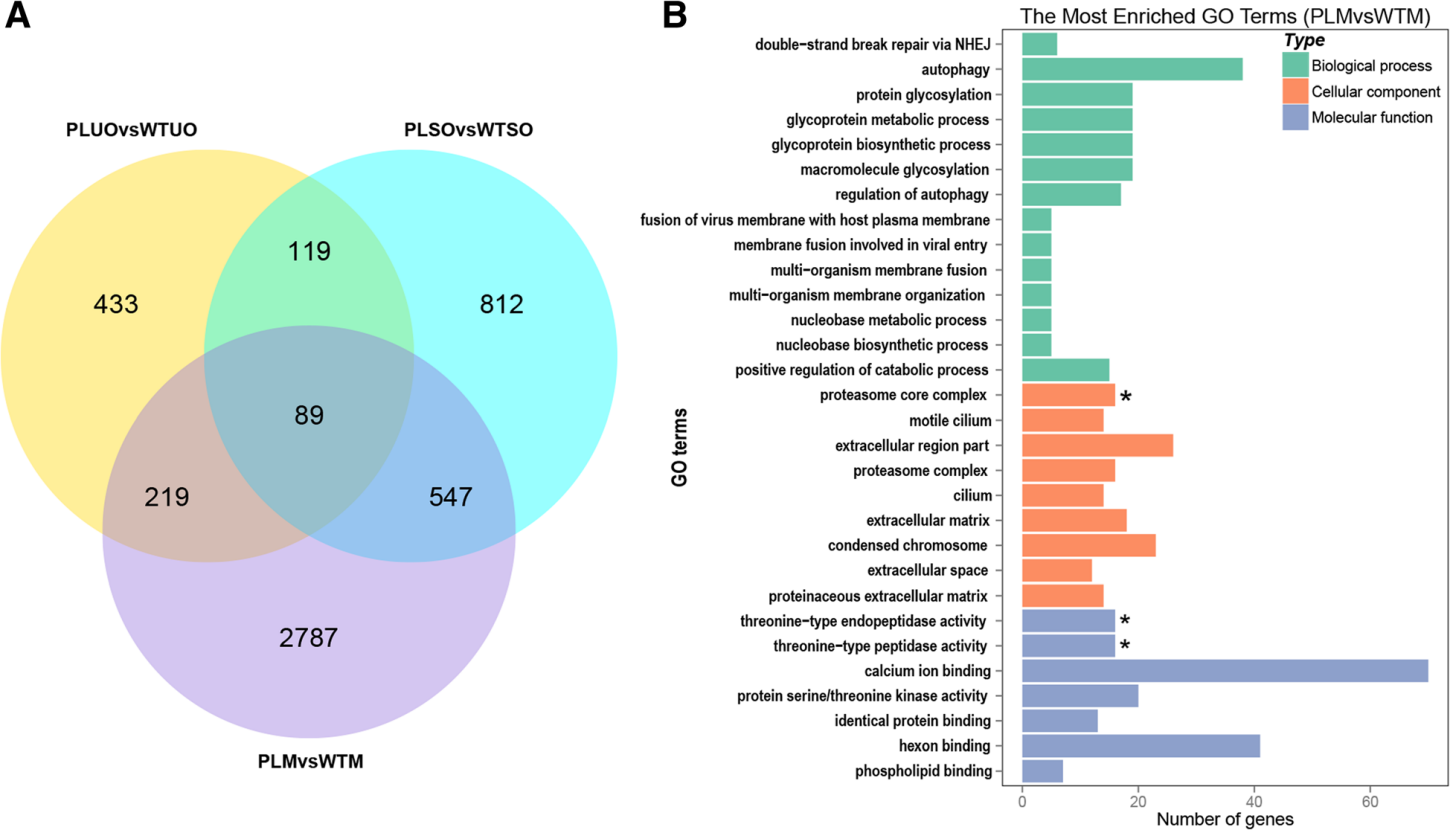
DEGs between *E. maxima* precocious line and its parent stain. **a**: Venn diagram of DEGs for three different stages of the *E. maxima* precocious line and its parent strain. Three different stages of *E. maxima* precocious line and its parent strain were collected for RNA-seq, and the DEGs between the same stages of the two line/strain were used for drawing Venn diagrams. **b**: Gene ontology enrichment of DEGs between merozoite stages of precocious line and its parent strain. WTSO vs PLSO, WTUO vs PLUO and WTM vs PLM represent DEGs between sporulated oocysts, unsporulated oocysts, and merozoites of BJ-WT-130 and BJ-PL-98. Asterisks represent statistically significant (*P* < 0.05)

In the sporulated oocyst stage, we found 1567 DEGs (Fig. 4a, Additional file 7). The top two enriched GO terms for these genes were structural constituent of ribosome and rough endoplasmic reticulum, but the result was not statistically significant. However, by searching within all 1567 DEGs annotations, we found that 10 eukaryotic translation initiation factors, three transcription elongation factors and many other molecules (including CDPKs, MICs, myosins and SAGs) involved in invasion were all highly transcribed in BJ-PL-98 (Additional file 8).

**Fig. 5:**
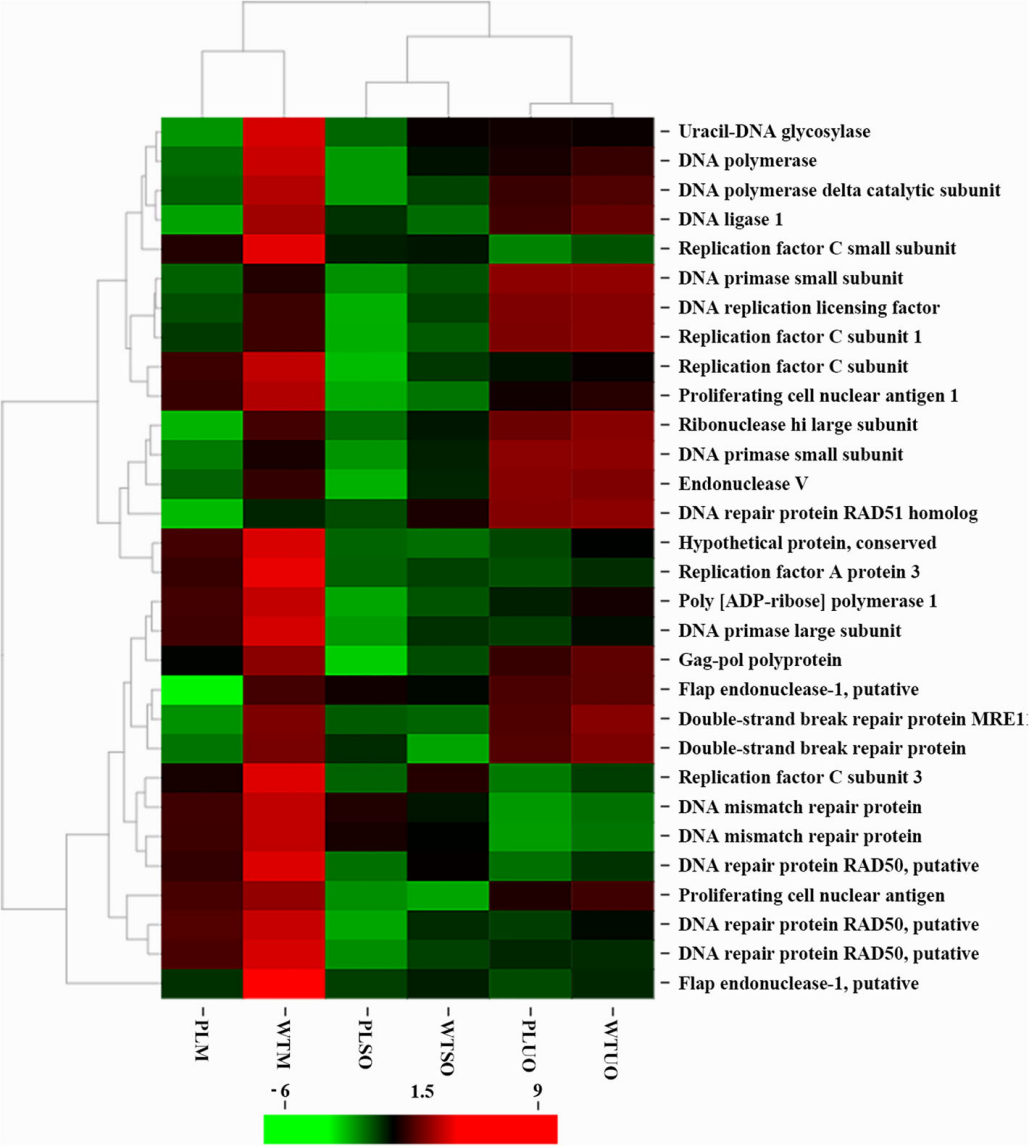
Clustered heatmap for DNA replication related genes. The mean log_2−_ FPKM value of every gene in each group was normalized and used. The R package pheatmap-based on Euclidean distance and complete clustering was used for the analysis. WTM and PLM: merozoites of BJ-WT-130 and BJ-PL-98, respectively; WTUO and PLUO: unsporulated oocysts of BJ-WT-130 and BJ-PL-98, respectively; WTSO and PLSO: sporulated oocysts of BJ-WT-130 and BJ-PL-98, respectively

We detected 3642 DEGs between merozoites of precocious line and its parent strain, which were the most among the three stages studied (Fig. 4a, Additional file 7). By GO enrichment analysis, we found that these DEGs were significantly enriched in proteasome related processes (Fig. 4b, Additional file 8). Thirty-one genes involved in DNA replication and mismatch repair pathways were also downregulated in BJ-PL-98 merozoites (Fig. 5). Additionally, ninety-nine DEGs were common in all three stages of *E. maxima* (Fig. 4a), including 12 DEGs downregulated and 16 DEGs upregulated in all three stages of BJ-PL-98 (Additional file 8).

## Discussion

Attenuated live vaccines provide an alternative choice for controlling coccidiosis as drug resistance in *Eimeria* has increased and drug residues increasingly appear in the food supply when anticoccidial drugs are widely used. In this study, we developed an *E. maxima* precocious line from the *E. maxima* Beijing strain. This precocious line has a prepatent time of 98 h and reduced fecundity and pathogenicity, but maintains the high immunogenicity of the parental strain. The precocious line still could not provide any cross-protection to different geographic strains as the parent strain (data not shown). Transcriptome analysis revealed that highly expressed translation initiation factors and invading factors in the sporulated oocyst stage of the precocious line may contribute to its shortened prepatent time while the suppressed protein cleavage activities and DNA replication progresses in merozoites possibly result in its decreased fecundity.

McDonald et al. (1986) [15] were the first to select an *E. maxima* precocious line with a reduction of 13 h in prepatent time (from 120 h to 107 h). Correspondingly, gametocytes appeared ~ 12 h ahead of time, and they speculated that there might be one asexual generation deleted, but they did not investigate further. The precocious line selected in this study has a reduction of 32 h in prepatent time (from 130 h to 98 h), which suggests that the BJ-PL-98 has a faster endogenous development. In the RNA-Seq data, we found highly activated biological progress involved in protein translation, such as “translation initiation factors” in the sporulated oocyst stage of the precocious line. This might be a “power storage” strategy for the precocious line, preparing many proteins required in subsequent invasion, proliferation, and/or division processes. Additionally, we detected that many CDPKs, MICs, myosins and SAGs were upregulated in the sporulated oocyst stage of the precocious line. These genes have been reported to be associated with parasite invasion [32–36], which might give the precocious line advantages in subsequent parasitism. According to the results in our endogenous development study, the precocious line and the parent stain can both invade host cells and developed into schizonts as early as 32 hpi, so that the “power storage” may be prepared for the schizogony development. In addition, the time required for invasion for both the precocious line and the wild strain should be measured more precisely, so that we can confirm our hypothesis. As we have presented, genes were significantly more enriched in protein translation related progresses in the merozoite stage (this also happened in the 3rd merozoite stage of *E. necartrix* [37]) than in the sporulated oocyst stage. This result suggests that merozoites have very fast metabolisms as a result of the fast growth, division and interaction with the host environment. The parasites could benefit in the next developmental stage by using this “power” stored in the previous stage.

Since we found substantial changes between the precocious line and its parent strain during the endogenous development stages, we decided to compare the gene expression profiles of both precocious line and parent strain, during the same endogenous development stages. We attempted several times to isolate merozoites of the 1st and 2nd generation schizonts and gametocytes, but all failed due to the extensive host contamination (only 5–30% reads could be mapped to *E. maxima* genome). According to our endogenous development study, we found that the largest amount of merozoites than any other generation occurred at 88 h in the precocious line and at 120 h in the wild type strain (data not shown), perhaps corresponding to the fourth generation, which was isolated for RNA seq. Based on our experimental methodology, we were unable to avoid the inclusion of parasites from other merozoites generations, and the above times are currently the best time points for sampling. For future studies, we are currently looking for an improved method to isolate the precise generation of schizonts.

In the endogenous development of *Eimeria* species, merozoites been through fast asexual expansion, which was accompanied by DNA replication and nuclear division. Thirty-one DNA replication related genes were downregulated in the BJ-PL-98 merozoite stage compared to its parent strain, which suggested that the precocious line had suppressed activities in DNA replication in the merozoite stage. This might be responsible for the reduced production of merozoites and thus further oocyst output. We also found that the protein cleavage related GO terms (16 proteasome subunit genes were involved) were significantly downregulated in BJ-PL-98. However, proteins been degraded by these peptidases should be identified and validated, so that we can know the whole regulation pathway.

Transcriptional comparison of the precocious line and its corresponding parent strain was also previously reported for the *E. tenella* Nippon strain [18]. The authors found that expression of carbohydrate metabolism in the virulent strain was stronger than that in the sporozoite stage of the precocious line. They believed that the parent strains survive long before the invasion and invade actively/successfully into host cells, whereas proliferative processes appear to affect precocity. Compared to ours, these different results may be due to differences between the distinct species studied, and because the authors used a single sample and a single stage for RNA-Seq. For these reasons, the mentioned hypotheses regarding the mechanisms of *Eimeria* precocity, based on high-throughput sequencing data, should be validated by further experiments, using, for example, in vitro culture models [38] or Cas9-based genetic manipulation.

## Conclusions

In this study, we developed a precocious line of *E. maxima* with a shortened prepatent time (from 130 h to 98 h). Comparative transcriptome analysis showed suppressed DNA replication activities in the merozoite stage in the precocious line, which may result in its reduced fecundity. Our study provides useful information to advance further studies in early gametocytogenesis in *Eimeria* species, as well as in other apicomplexan parasites.

## Additional files

Additional file 1: Details of *E. maxima* precocious line selection. (DOCX 36 kb)

Additional file 2: Primers used in qPCR experiments. (DOCX 16 kb)

Additional file 3: Comparative endogenous development for the *E. maxima* wild strain and its precocious line. Chickens were sacrificed after indicated times post-inoculation with the *E. maxima* wild strain BJ-WT-130 and the precocious line BJ-PL-98. The small intestines were used for H&E staining. Black, white and red arrows indicate the schizonts, gametocytes and unsporulated oocysts, respectively. Black arrowhead indicates microgametocytes. Bar = 20 μm. (PDF 52347 kb)

Additional file 4: Statistics of RNA-seq data mapped to *E.maxima* genome. Genome and gene models of *E. maxima* in ToxoDB release-35 was downloaded for analysis. (DOCX 24 kb)

Additional file 5: Clustered heatmap of all DEGs in three stages of the precocious line and its parent strain. WTM and PLM: merozoites of BJ-WT-130 and BJ-PL-98, respectively; WTUO and PLUO: unsporulated oocysts of BJ-WT-130 and BJ-PL-98, respectively; WTSO and PLSO: sporulated oocysts of BJ-WT-130 and BJ-PL-98, respectively. (PDF 2502 kb)

Additional file 6: qPCR validation of six genes in different stages of the *E. maxima* precocious line and its parent strain. Gene expression was normalized to the reference gene GAPDH; asterisks represent *p* < 0.01. (TIF 331 kb)

Additional file 7: Mean-abundance (MA) plots and Volcano plots comparisons of DEGs between BJ-WT-130 and BJ-PL-98 in three different development stages. (PDF 1425 kb)

Additional file 8: Detailed DEGs of different comparison groups between the *E. maxima* wild strain and its precocious line. (XLSX 152 kb)
