# Supplementary material for: Comparative transcriptome analysis of Eimeria maxima (Apicomplexa: Eimeriidae) suggests DNA replication activities correlating with its fecundity
Source: BMC Genomics. 2018 Sep 24;19:699. doi: 10.1186/s12864-018-5090-2 (PMC6154952; doi:10.1186/s12864-018-5090-2)
Supplement: Supplementary file 1 — Details of E. maxima precocious line selection. (DOCX 36 kb) [file 12864_2018_5090_MOESM1_ESM.docx]

Additional file 1：Details for *E.maxima* precocious line selection.

| Generations | Birds  (day) | Inoculation dose | Oocyst collection time  (h.p.i.) | Oocyst yield | Prepatent time  (h.p.i.) |
| --- | --- | --- | --- | --- | --- |
| P1 | 4（8） | 5000 | 121-133 | 2.43×10^6^ | 130 |
| P2 | 4（24） | 6000 | 131-139 | 9.0×10^5^ |  |
| P3 | 5（6） | 5000 | 121-125 | 1000 |  |
| P4 | 4（18） | 100 | 129-148 | 3.0×10^6^ |  |
| P5 | 5（5） | 5000 | 122-127.5 | 4.0×10^6^ |  |
| P6 | 3（18） | 1500 | 126-142 | 1.2×10^5^ |  |
| P7 | 4（27） | 25000 | 119-132 | 4.0×10^6^ |  |
| P8 | 5（4） | 30000 | 117-127 | 1.2×10^6^ |  |
| P9 | 5（11） | 5000 | 107-140 | 8.0×10^3^ |  |
| P10 | 2（15） | 10000 | 114-142 | 3000 |  |
| P11 | 3（24） | 1000 | 103-136 | 2.5×10^6^ |  |
| P12 | 2（32） | 5000 | 110-136 | 1.75×10^5^ |  |
| P13 | 2（40） | 65000 | 110-130 | 3.75×10^5^ |  |
| P14 | 3（10） | 10000 | 105-138 | 2.5×10^4^ |  |
| P15 | 4（20） | 5000 | 108-136 | 7.2×10^4^ |  |
| P16 | 4（30） | 15000 | 110-137 | 5.0×10^4^ |  |
| P17 | 3（38） | 15000 | 108-140 | 1.0×10^5^ |  |
| P18 | 4（20） | 20000 | 108-137 | 1.9×10^6^ |  |
| P19 | 5（8） | 100000 | 108-132 | 3.0×10^6^ |  |
| P20 | 5（17） | 180000 | 100-120 | 3.5×10^6^ | 108 |
| P21 | 4（27） | 350000 | 96-112 | 4.2×10^5^ |  |
| P22 | 6（7） | 70000 | 96-107 | 4.1×10^6^ | 98 |
| P23 | 4（19） | 100000 | 84-102 | 4.2×10^4^ | 98 |
| P24 | 4（7） | 8000 | 84-106 | 1.35×10^4^ | 98 |
| P25 | 3（14） | 4000 | 84-109 | 4×10^4^ | 98 |

P0：BJ-WT-130; P25: BJ-PL-98
