# Supplementary material for: Comparative transcriptome analysis of Eimeria maxima (Apicomplexa: Eimeriidae) suggests DNA replication activities correlating with its fecundity
Source: BMC Genomics. 2018 Sep 24;19:699. doi: 10.1186/s12864-018-5090-2 (PMC6154952; doi:10.1186/s12864-018-5090-2)
Supplement: Supplementary file 2 — Primers used in qPCR experiments. (DOCX 16 kb) [file 12864_2018_5090_MOESM2_ESM.docx]

**Additional file 2：**

Table: Primers used in qPCR experiments.

| Primers | 5’-3’ |
| --- | --- |
| EMWEY_00029960-F | TGGCCTGTTGAATTTGGGGA |
| EMWEY_00029960-R | GCTTGCCGAACCAAACAGAG |
| EMWEY_00045780-F | AATGAAGTCCGCAGCCAAGA |
| EMWEY_00045780-R | GCCCTTCTTCCATTCGGTGA |
| EMWEY_00048100-F | CCTGGCAACTCGCTTCAGTA |
| EMWEY_00048100-R | TCGTTGGTGGGGATCGAAAG |
| EMWEY_00018530-F | CCTGAGGCGGTTGTTCTTCA |
| EMWEY_00018530-R | AGGTGTATCCTCCACCACCA |
| EMWEY_00040120-F | CCAGAAGCCAGAAAATGCCC |
| EMWEY_00040120-R | CCCATCCTTGCGACCTGAAT |
| EMWEY_00032200-F | GACAGACGAGGCGAAAGACA |
| EMWEY_00032200-R | AGAAGCAACGGATAGCCACC |
| GAPDH-F | ATGTGTTCGCCGAGAAGGAG |
| GAPDH-R | GACCACCTGGAGAGAGGACT |
