# Supplementary figures and images for: Comparative transcriptome analysis of Eimeria maxima (Apicomplexa: Eimeriidae) suggests DNA replication activities correlating with its fecundity
Source: BMC Genomics. 2018 Sep 24;19:699. doi: 10.1186/s12864-018-5090-2 (PMC6154952; doi:10.1186/s12864-018-5090-2)

**BJ-WT-130****BJ-PL-98****32h**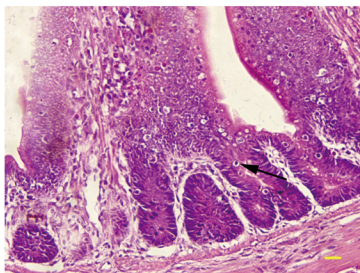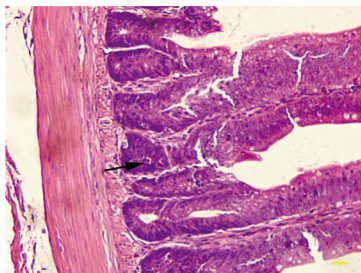**64h**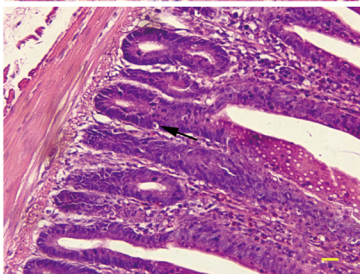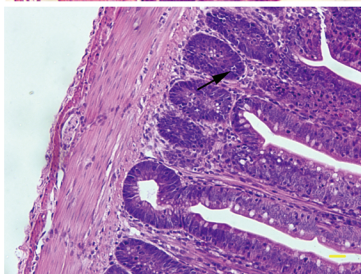**80h**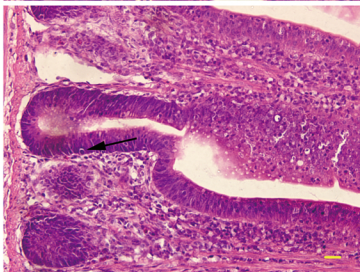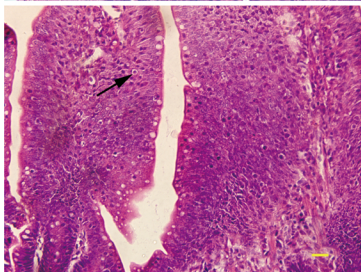**88h**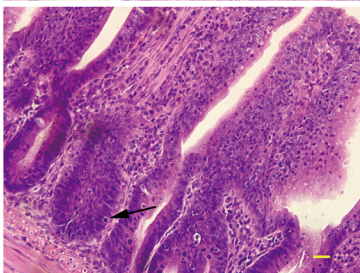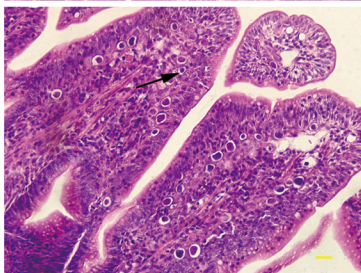**96h**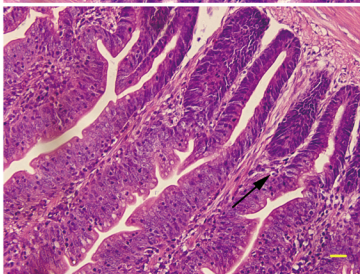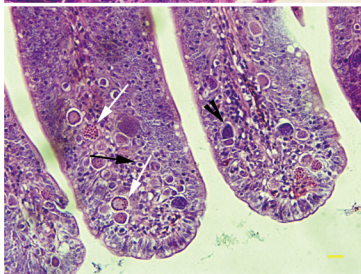**104h**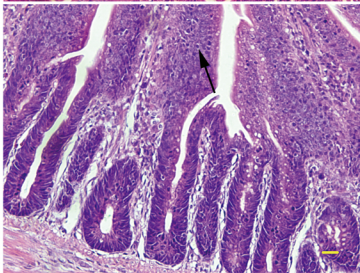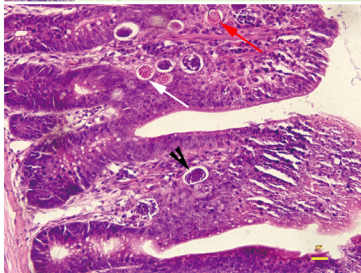

**BJ-WT-130****BJ-PL-98****112h**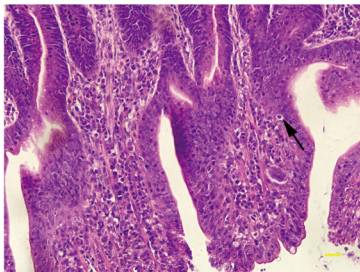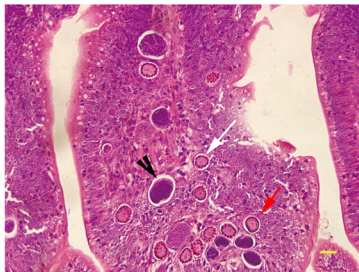**120h**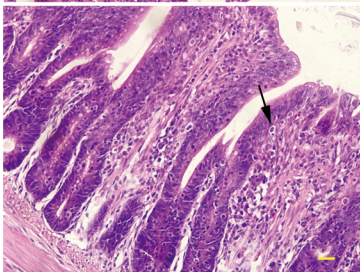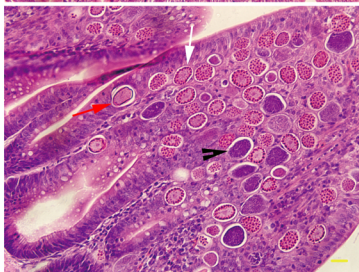**128h**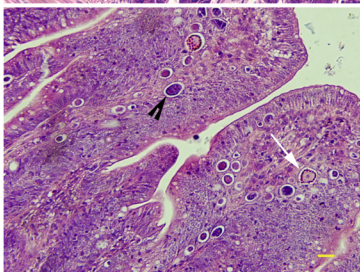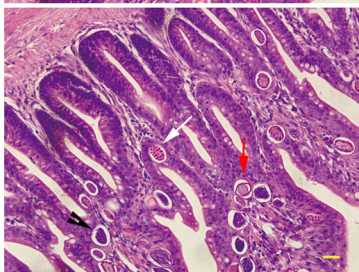**140h**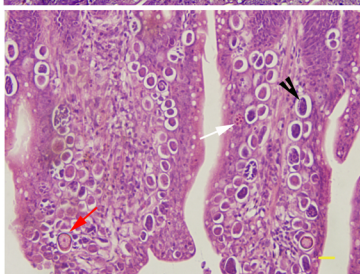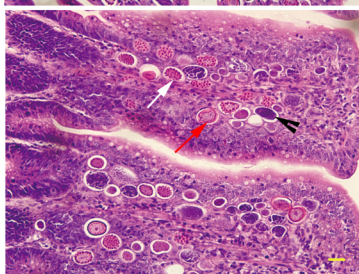**150h**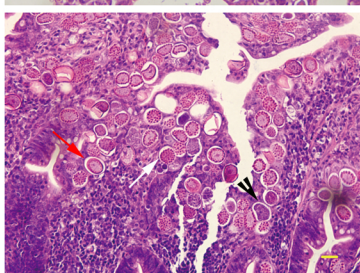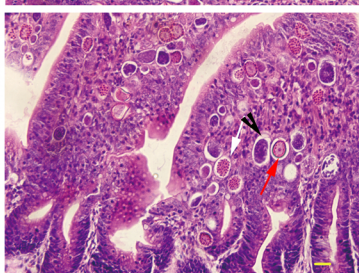

Supplement: Supplementary file 3 — Comparative endogenous development for the E. maxima wild strain and its precocious line. Chickens were sacrificed after indicated times post-inoculation with the E. maxima wild strain BJ-WT-130 and the precocious line BJ-PL-98. The small intestines were used for H&E staining. Black, white and red arrows indicate the schizonts, gametocytes and unsporulated oocysts, respectively. Black arrowhead indicates microgametocytes. Bar = 20 μm. (PDF 52347 kb) [file 12864_2018_5090_MOESM3_ESM.pdf]

## Cluster analysis of differentially expressed genes

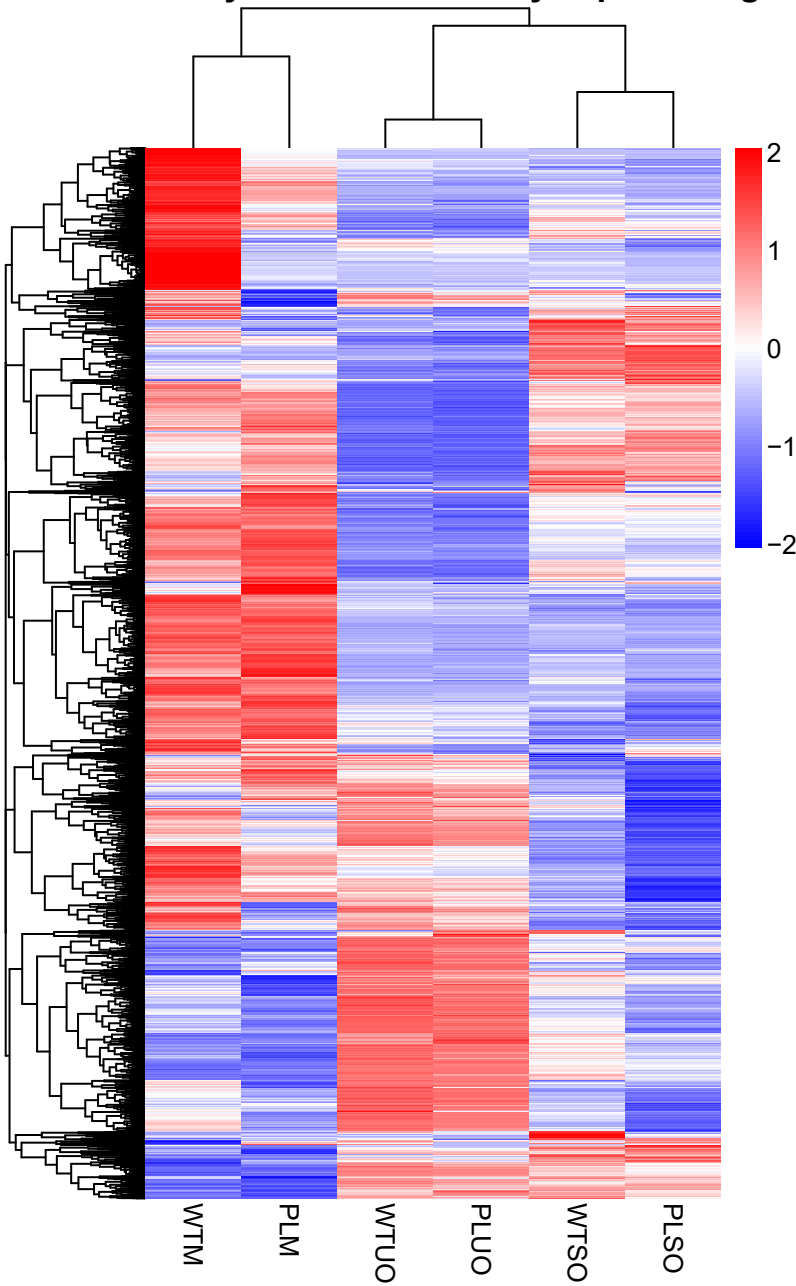

Supplement: Supplementary file 5 — Clustered heatmap of all DEGs in three stages of the precocious line and its parent strain. WTM and PLM: merozoites of BJ-WT-130 and BJ-PL-98, respectively; WTUO and PLUO: unsporulated oocysts of BJ-WT-130 and BJ-PL-98, respectively; WTSO and PLSO: sporulated oocysts of BJ-WT-130 and BJ-PL-98, respectively. (PDF 2502 kb) [file 12864_2018_5090_MOESM5_ESM.pdf]

PLUO vs WTUO

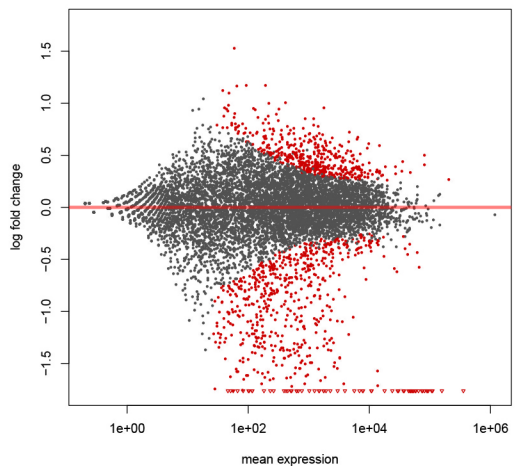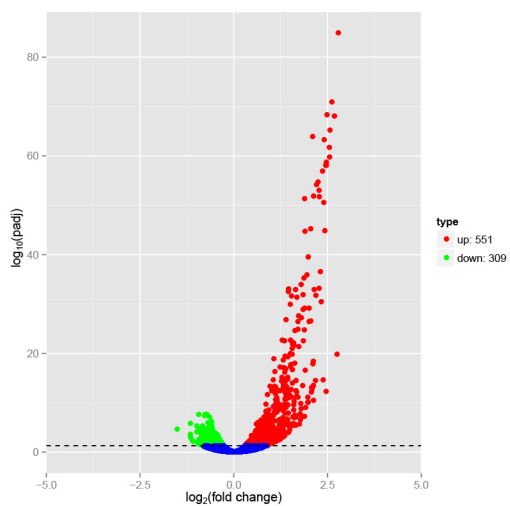

PLSO vs WTSO

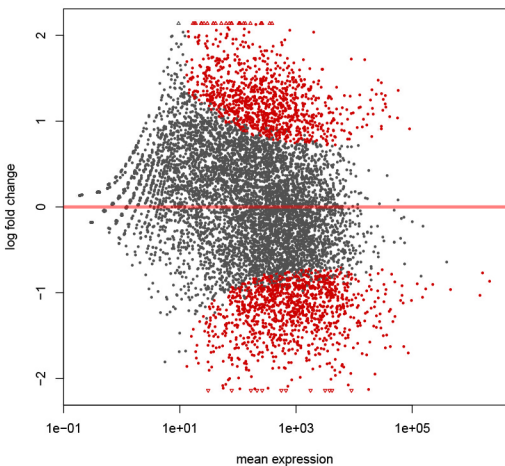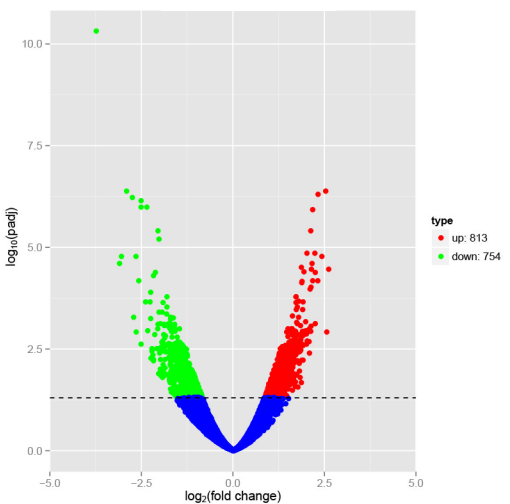

PLM vs WTM

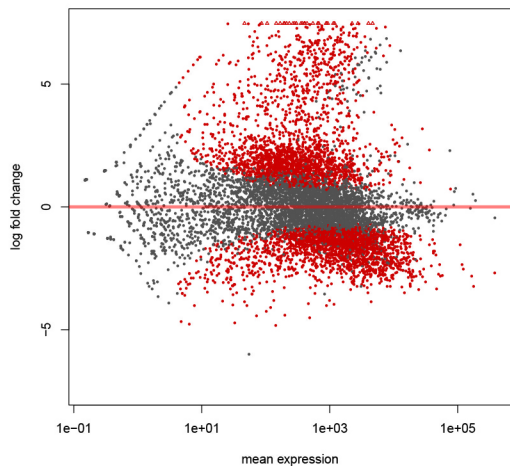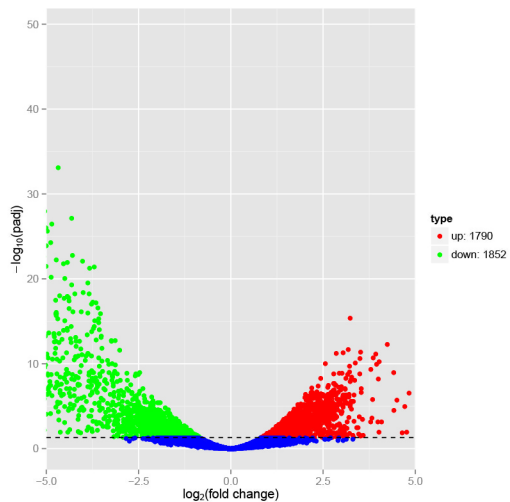

Supplement: Supplementary file 7 — Mean-abundance (MA) plots and Volcano plots comparisons of DEGs between BJ-WT-130 and BJ-PL-98 in three different development stages. (PDF 1425 kb) [file 12864_2018_5090_MOESM7_ESM.pdf]
