# Supplementary material for: Comparative transcriptome analysis of Eimeria maxima (Apicomplexa: Eimeriidae) suggests DNA replication activities correlating with its fecundity
Source: BMC Genomics. 2018 Sep 24;19:699. doi: 10.1186/s12864-018-5090-2 (PMC6154952; doi:10.1186/s12864-018-5090-2)
Supplement: Supplementary file 4 — Statistics of RNA-seq data mapped to E.maxima genome. Genome and gene models of E. maxima in ToxoDB release-35 was downloaded for analysis. (DOCX 24 kb) [file 12864_2018_5090_MOESM4_ESM.docx]

| **Sample_name** | **Em_WT_M1** | **Em_WT_M2** | **Em_PL_M3** | **Em_PL_M1** | **Em_PL_M2** | **Em_WTUO1** | **Em_WTUO2** | **Em_WTSO1** | **Em_WTSO2** | **Em_PLUO1** | **Em_PLUO2** | **Em_PLSO3** | **Em_PLSO4** |
| --- | --- | --- | --- | --- | --- | --- | --- | --- | --- | --- | --- | --- | --- |
| Total reads | 56243438 | 59874364 | 47322938 | 51686396 | 47129204 | 68923232 | 60549916 | 52308050 | 49381710 | 56168990 | 55410128 | 47937610 | 47703434 |
| Total mapped | 41858699 (74.42%) | 44556396 (74.42%) | 38267931 (80.87%) | 39539193 (76.5%) | 33568873 (71.23%) | 56061963 (81.34%) | 48776701 (80.56%) | 44115610 (84.34%) | 39913216 (80.83%) | 47184247 (84%) | 46739424 (84.35%) | 41644676 (86.87%) | 41532347 (87.06%) |
| Multiple mapped | 324624 (0.58%) | 335273 (0.56%) | 302230 (0.64%) | 309997 (0.6%) | 280546 (0.6%) | 638186 (0.93%) | 575416 (0.95%) | 651337 (1.25%) | 486789 (0.99%) | 680203 (1.21%) | 612955 (1.11%) | 446614 (0.93%) | 488194 (1.02%) |
| Uniquely mapped | 41534075 (73.85%) | 44221123 (73.86%) | 37965701 (80.23%) | 39229196 (75.9%) | 33288327 (70.63%) | 55423777 (80.41%) | 48201285 (79.61%) | 43464273 (83.09%) | 39426427 (79.84%) | 46504044 (82.79%) | 46126469 (83.25%) | 41198062 (85.94%) | 41044153 (86.04%) |
| Reads map to '+' | 20716976 (36.83%) | 22057884 (36.84%) | 18942487 (40.03%) | 19575791 (37.87%) | 16619297 (35.26%) | 27560005 (39.99%) | 23960666 (39.57%) | 21575826 (41.25%) | 19634847 (39.76%) | 23145678 (41.21%) | 22945981 (41.41%) | 20508036 (42.78%) | 20431143 (42.83%) |
| Reads map to '-' | 20817099 (37.01%) | 22163239 (37.02%) | 19023214 (40.2%) | 19653405 (38.02%) | 16669030 (35.37%) | 27863772 (40.43%) | 24240619 (40.03%) | 21888447 (41.85%) | 19791580 (40.08%) | 23358366 (41.59%) | 23180488 (41.83%) | 20690026 (43.16%) | 20613010 (43.21%) |
| Non-splice reads | 29910943 (53.18%) | 31838171 (53.17%) | 29213673 (61.73%) | 29645260 (57.36%) | 25102105 (53.26%) | 42249053 (61.3%) | 36644954 (60.52%) | 35512194 (67.89%) | 31281218 (63.35%) | 33138263 (59%) | 32970449 (59.5%) | 34110772 (71.16%) | 34215566 (71.73%) |
| Splice reads | 11623132 (20.67%) | 12382952 (20.68%) | 8752028 (18.49%) | 9583936 (18.54%) | 8186222 (17.37%) | 13174724 (19.12%) | 11556331 (19.09%) | 7952079 (15.2%) | 8145209 (16.49%) | 13365781 (23.8%) | 13156020 (23.74%) | 7087290 (14.78%) | 6828587 (14.31%) |

Additional file 4: statistics of RNA-seq data mapped to *E.maxima* genome.
